# HIV-1 replication and latency are balanced by mTOR-driven cell metabolism

**DOI:** 10.3389/fcimb.2022.1068436

**Published:** 2022-11-17

**Authors:** Jacqueline M. Crater, Douglas F. Nixon, Robert L. Furler O’Brien

**Affiliations:** Division of Infectious Diseases, Department of Medicine, Weill Cornell Medicine, New York, NY, United States

**Keywords:** HIV-1, immunometabolism, CD4 + T cell, mTOR, glycolysis

## Abstract

Human Immunodeficiency virus type 1 (HIV-1) relies on host cell metabolism for all aspects of viral replication. Efficient HIV-1 entry, reverse transcription, and integration occurs in activated T cells because HIV-1 proteins co-opt host metabolic pathways to fuel the anabolic requirements of virion production. The HIV-1 viral life cycle is especially dependent on mTOR, which drives signaling and metabolic pathways required for viral entry, replication, and latency. As a central regulator of host cell metabolism, mTOR and its downstream effectors help to regulate the expression of enzymes within the glycolytic and pentose phosphate pathways along with other metabolic pathways regulating amino acid uptake, lipid metabolism, and autophagy. In HIV-1 pathogenesis, mTOR, in addition to HIF-1α and Myc signaling pathways, alter host cell metabolism to create an optimal environment for viral replication. Increased glycolysis and pentose phosphate pathway activity are required in the early stages of the viral life cycle, such as providing sufficient dNTPs for reverse transcription. In later stages, fatty acid synthesis is required for creating cholesterol and membrane lipids required for viral budding. Epigenetics of the provirus fueled by metabolism and mTOR signaling likewise controls active and latent infection. Acetyl-CoA and methyl group abundance, supplied by the TCA cycle and amino acid uptake respectively, may regulate latent infection and reactivation. Thus, understanding and exploring new connections between cellular metabolism and HIV-1 pathogenesis may yield new insights into the latent viral reservoirs and fuel novel treatments and cure strategies.

## Introduction

Despite therapeutic advancements which control HIV-1 viral loads in people living with HIV (PLWH), a widespread cure for HIV-1 remains elusive due to the establishment of latent and persistent viral reservoirs within CD4^+^ receptor-bearing T cells and macrophages. CD4^+^ T cells are markedly depleted over the course of untreated HIV-1 disease and are often considered the primary target of HIV-1 infection (Levy, 1993).

HIV-1 replicates more productively in activated CD4^+^ T cells which have increased nutrient uptake and aerobic glycolysis, a metabolic shift in highly proliferative cells known as the Warburg effect (Hollenbaugh et al., 2011; Maciolek et al., 2014; Hegedus et al., 2014). Although indicative of many cancer cells, activated lymphocytes also undergo this metabolic switch to increase glucose uptake and lactate production even in the presence of oxygen (Maciolek et al., 2014). The metabolic reprogramming seen in activated T cells is regulated largely by mammalian target of rapamycin (mTOR) signaling. mTOR associates with additional host proteins to create two complexes, mTORC1 and mTORC2, which are central signaling regulators of cellular metabolism (Ben-Sahra and Manning, 2017). mTOR’s upstream and downstream signaling molecules are altered by HIV-1 infection to regulate the viral life cycle and latency (Besnard et al., 2016), which will be a central theme in this review.

Physiological mTOR signaling affects T cell subset differentiation, which is explained in depth in a review from Huang et al. (Huang et al., 2020). mTORC1 signaling promotes Th1 differentiation *via* STAT4/SOCS3 and Th17 differentiation *via* HIF-1α (Huang et al., 2020). While mTORC2 is necessary for Th2 differentiation, both mTOR complexes are needed for Tfh differentiation (Huang et al., 2020). Mice deficient in Rictor, a necessary subunit of mTORC2, showed reduce Tfh differentiation (Zeng et al., 2016). Rictor-deficient mice also showed decreased memory phenotype CD4^+^ T cells in Peyer’s patches when compared to Raptor(mTORC1) deficient mice (Zeng et al., 2016). Treg differentiation occurs at when there is decreased activity of both mTORC1 and mTORC2 (Huang et al., 2020). Although many of the T cell studies linking mTOR activity to cellular differentiation were done in animal models, mTORC1 and mTORC2 activity levels and timing are likely to influence human T cell proliferation, differentiation, and migration.

mTOR and related phosphorylation cascades regulate downstream metabolic pathways that are critical for viral replication. These metabolic pathways including glycolysis, the pentose phosphate pathway, tricarboxylic acid (TCA) cycle, glutaminolysis, and oxidative phosphorylation will be discussed both in the context of physiological T cell function and during HIV-1 replication and latency. While this review focuses on the importance of cellular metabolism on HIV replication and latency, there are several in-depth reviews that cover physiological metabolic pathways within uninfected T cells and macrophages (MacIver et al., 2013; Zhu et al., 2015).

This review will also primarily address experiments done in human primary cells. Although several reports use models of HIV-1 replication in cell lines such as Jurkats, these results are difficult to compare to physiological metabolic activity within primary CD4^+^ T cells. Cells lines often have elevated expression of oncogenes, decreased levels of tumor repressor genes, exhibit continuous cycling and proliferation, and often contain an irregular number of chromosomes, resulting in different innate metabolic profiles including higher baseline glycolytic activity. For example, Jurkat cell lines contain elevated PIP_3_ levels due to a defective PTEN phosphatase, which induces a hyperactivation of AKT, mTORC1, and greatly influences downstream metabolic pathways (Gioia et al., 2018). Results of metabolic studies obtained in cell line cultures should be cautiously interpreted in light of these differences. In this review, results gathered from cell lines and animal models are only used when no primary human cell data has been reported.

The relationship between HIV-1 replication and immunometabolism has been a quickly growing field but remains a topic in which additional research and scrutiny are needed. A recent 2021 review from Sáez-Cirión et al. discusses the metabolism of CD8^+^ T cell responses to HIV-1 infected cells (Saez-Cirion and Sereti, 2021). They expand on chronic inflammation in HIV-1 infection as well as CD8^+^ T cell exhaustion and metabolic dysfunction. A much richer history of T cell metabolism research exists than what is presented in this review and should be explored.

This review will begin with the signaling pathways that regulate CD4^+^ T cell metabolism and their role in HIV-1 pathogenesis. Then, perturbations of specific downstream metabolic pathways during HIV-1 infection will be discussed, followed by the relationship of metabolism to epigenetics and HIV-1 latency. To conclude, the metabolic requirements for each stage of the HIV-1 life cycle will be discussed.

## HIV-1 dependence on metabolically linked signaling pathways

The flux between catabolic and anabolic pathways in a living cell is under the dynamic control by multiple signaling pathways regulated by mTOR. These signaling pathways are exploited by HIV-1 during its replication cycle. Although linkages between these signaling cascades and metabolic pathways in uninfected T cells have been reviewed elsewhere (Saravia et al., 2020), this section will review how signaling pathways that regulate the metabolic reprogramming of cells affect HIV-1 replication, with mTOR signaling as a central hub.

### Physiological activation of mTOR and its requirement for HIV-1 replication

Metabolic requirements of a T cell change throughout its lifecycle. Cell division, migration, and differentiation are intricately regulated by available metabolites along with the enzymes and signaling pathways that govern them. A central regulator of cell metabolism is mTOR, which can form two complexes that balance the dynamic metabolic needs of a living cell (Magaway et al., 2019), and mTOR activity has been found to be increased in CD4^+^ T cells of viremic PLWH (Taylor et al., 2020). The mTOR complexes integrate multiple intracellular or extracellular signals to regulate physiological T cell metabolsim and HIV-1 replication. mTOR can be activated by extracellular signals that stimulate the upstream PI3K/AKT pathway but mTOR signaling is also regulated by intracellular metabolite levels including nucleotides and amino acids. When the intracellular levels of energy-rich nutrients declines, mTOR can regulate autophagy or affect cell migration through mTORC2-regulated cytoskeletal remodeling (Dai and Thomson, 2019). AMP/ADP/ATP levels affect AMPK activity, which directly and indirectly affect mTOR activation. The mTORC1 complex is also inhibited during times of decreased amino acid availability (Hara et al., 1998; Ma and Blenis, 2009; Inoki et al., 2012). mTORC1 is activated by increased glutamine levels and subsequent glutaminolysis (Durán et al., 2012). Glutamine utilization is correlated to both HIV-1 infection susceptibility and to late stage virion production (Hegedus et al., 2017).

When a T cell is activated through its T cell receptor (TCR) or *via* CD3 and co-stimulatory molecule CD28, the T cell’s metabolic requirements shift and an influx of nutrients from the environment enters the cell. T cell stimulation activates the PI3K and Akt pathways, which are upstream regulators of mTOR that affect cell metabolism and glucose uptake that drive anabolic pathways required for cell proliferation (Zhang et al., 2011; Courtnay et al., 2015). PI3K activation leads to the phosphorylation of Akt and subsequently increases the activity of mTORC1. This increased mTORC1 activity reprograms the metabolic profile of T cells to favor increased ATP production through aerobic glycolysis (Maciolek et al., 2014), increased protein synthesis through S6K1 and 4E-BP1 phosphorylation (Ma and Blenis, 2009; Ben-Sahra and Manning, 2017), increased lipid and nucleic acid anabolism through induction of the Pentose Phosphate Pathway (Ben-Sahra and Manning, 2017), and increased glutamine utilization through downstream HIF-1α and Myc-mediated transcription (Magaway et al., 2019).

HIV-1 entry and replication requires PI3K/Akt/mTOR signaling. HIV-1 Env (gp120) activates the PI3K/Akt pathway in PM1 and TZM-bl cell lines following binding to the CD4 molecule. Viral entry is blocked when PTEN, a PI3K/Akt pathway inhibitor, is overexpressed in target cells (Hamada et al., 2019). HIV NL4.3 Env/gp120 induces AKT phosphorylation in infected primary CD4^+^ T cells relative to uninfected or NL4.3ΔEnv infected primary CD4^+^ T cells (Hegedus et al., 2014). mTOR inhibition has further been shown to prevent CCR5-tropic viral entry to a greater degree in primary CD4+ T cells than entry of CXCR4-tropic viruses (Heredia et al., 2015). HIV-1 Env-mediated upregulation of mTOR signaling has also been reported in dendritic cells (DC) (Blanchet et al., 2010). Exposure of DCs to HIV-1 for 15 minutes induced phosphorylation of Erk, upstream of mTOR, and S6, downstream of mTOR. Exposure of DCs to HIV-1 ΔEnv did not cause this upregulation. The increase in mTOR signaling in DCs led to a block in autophagy initiation, which was rescued with the addition of rapamycin (Blanchet et al., 2010).

Following entry, productive HIV-1 replication is also inhibited in primary CD4^+^ T cells when PI3K/Akt/mTOR signaling activity is decreased (Taylor et al., 2020). Primary CD4^+^ T cells were more permissive to productive HIV-1 replication following Akt activation and subsequent upregulation of GLUT1, glucose influx, and aerobic glycolysis (Loisel-Meyer et al., 2012; Courtnay et al., 2015). mTOR activation also positively correlated with HIV-1 expression using a primary human CD4^+^ T cell latency model (Besnard et al., 2016). mTOR activity is upregulated 24 hours post-infection in PBMC cultures (Kumar et al., 2017), perhaps due to interactions between HIV-1 Nef and Akt (Kumar et al., 2016).

HIV transcription and latency is also linked to mTOR’s regulation of metabolites required for epigenetic modifications including aceytl-CoA and substrates for one-carbon metabolism. mTOR alters intracellular levels of acetyl-CoA through the activation of Akt and ATP citrate lyase (Taylor et al., 2020). When HIV-1 is reactivated from latency in primary cell models, the mTORC1 pathway is upregulated in CD4^+^ T cells. Glycolytic enzymes downstream of mTORC1 that affect acetyl-CoA levels, such as LDHA, HK2, and GAPDH, are increased during reactivation (Dobrowolski et al., 2019). Using a J-LAT 5A8 cell line, which contains an integrated reporter virus to model HIV-1 latency and reactivation, Besnard et al. completed an shRNA screen followed by chemical inhibition experiments to link mTORC1/2 components as critical regulators of HIV-1 transcription (Besnard et al., 2016).

### HIF-1α activity and HIV-1 replication

HIF-1α, a subunit of HIF-1, is a downstream transcription factor of mTORC1 and is involved in the homeostatic cellular response to hypoxia (Semenza, 2009). Under normoxic conditions, HIF-1α is degraded by the proteosome, but under sustained hypoxic conditions, HIF-1α dimerizes with HIF-1β (Semenza, 2009) and induces transcription of genes that regulate glucose metabolism including pyruvate dehydrogenase kinase 1 (Semenza, 2009), GLUT1, aldolase A, PGK1, pyruvate kinase M, and lactate dehydrogenase A (Semenza et al., 1994; Luo et al., 2011).

HIV-1 infection increases oxidative stress within the cell (Legrand-Poels et al., 1990; Sandstrom et al., 1993), which leads to an increase in HIF-1α expression and activity (Land and Tee, 2007; Duette et al., 2018). This state of oxidative stress allows HIF-1α to translocate into the nucleus and glycolytic genes to be transcribed. HIF-1α was expressed in HIV-1 infected primary CD4^+^ T cells in higher levels than mock infected cells 48 hours post infection. Interestingly, bystander uninfected CD4^+^ T cells were also found to have slightly increased HIF-1α expression (Duette et al., 2018), possibly due to the uptake of extracellular HIV-1 Tat. Infected cells showed increased levels of GLUT1 and glucose uptake. This influx of glucose is metabolized by higher enzymatic levels of HK1 and LDH within infected cells. HIV-1 infection increases glycolysis and fermentation to elevate the cellular extracellular acidification rate (ECAR), which is an indicator of glycolysis. The ECAR found in these infected cells correlated with increased HIF-1α activity (Duette et al., 2018). In contrast, cells treated with an HIF-1α inhibitor showed decreased expression of proteins involved in aerobic glycolysis (Duette et al., 2018). However, antiretrovial therapy (ART) may not fully restore HIF-1α activity to baseline following infection. Quiescent CD4^+^ T cells from PLWH on ART were additionally found to have significantly higher levels of HIF-1α expression than HIV-uninfected individuals, which correlated with surface GLUT1 abundance (Duette et al., 2018).

In contrast to the effects of HIF-1α described, HIF-2α has been found to repress transcription at the HIV-1 promotor (Zhuang et al., 2020). Primary CD4^+^ T cells cultured in a physiological 1% oxygen environment, as standard for lymph nodes, rather than 20%, as standard for cell cultures, showed a 50% decrease in LTR activity without decreasing viability or activation of the cells (Zhuang et al., 2020). Jurkats cultured in 1% oxygen with a knockout of HIF-1α showed a slight decrease in LTR activity, while HIF-2α knockout Jurkats in 1% oxygen showed over a 2-fold increase in LTR transcription (Zhuang et al., 2020). This indicates that HIF-2α, rather than HIF-1α, is responsible for LTR silencing in hypoxic conditions. This work in cell lines will need to be verified using more physiological conditions.

### Myc activity and HIV-1 replication

Myc is another transcription factor critical for T cell metabolism (Wang et al., 2011). Myc works in parallel to the PI3K/Akt/mTOR and HIF-1α signaling cascades and is also utilized by HIV-1. Targets of Myc and glycolytic enzymes are upregulated upon reactivation from HIV-1 latency in CD4^+^ T cells (Dobrowolski et al., 2019). Myc has additionally been shown to be crucial for dNTP availability in HIV-1 infected primary CD4^+^ T cells, as well as the upregulation of GLUT1 and amino acid transporters such as ASCT2 required for HIV-1 virion production (Taylor et al., 2015).

The direct interaction between Myc and mTOR signaling is not fully understood, but inhibition of mTORC1 with rapamycin decreases both Myc and HIF-1α expression (Wang et al., 2011). Decreased Myc expression also leads to lower levels of glycolytic enzymes such as HK and PKM2 in T lymphocytes (Wang et al., 2011). This accompanies a slight decrease in pentose phosphate pathway activity and the decrease of Tkt and G6PDH enzymes (Wang et al., 2011). HIV-1 is known to be reliant on T cell activation (Hegedus et al., 2014; Valle-Casuso et al., 2019), indicating the importance of Myc for infectivity and virion production through the upregulation of glycolytic and PPP enzymes.

## HIV-1 infection alters metabolic pathways

HIV-1 virion production requires the same energy-consuming anabolic pathways that promote cell growth and proliferation following T cell activation. To increase biological mass, energy in the form of ATP is used to connect smaller building blocks into larger structures. For T cells to grow or proliferate, they require both a source of ATP and a source of building blocks which were not present at elevated levels during their quiescent state.

Elevated ATP production occurs through a dramatic influx of glucose following T cell activation. Although elevated oxidative phosphorylation (OXPHOS) also occurs following T cell activation, aerobic glycolysis is required to quickly produce higher levels of ATP while still replenishing the NAD^+^ supply required for glucose breakdown. T cell activation increases mitochondrial activity and OXPHOS which leads to increased production of reactive oxygen species (ROS). To compensate for potential oxidative damage due to elevated ROS, cells can route incoming glucose through the pentose phosphate pathway (PPP) to produce antioxidant-enhancing NADPH. Metabolites from the PPP and an influx of amino acids following T cell activation provides the necessary building blocks for nucleic acid, protein, lipid, and carbohydrate anabolism (Duvel et al., 2010).

**Figure 1:**
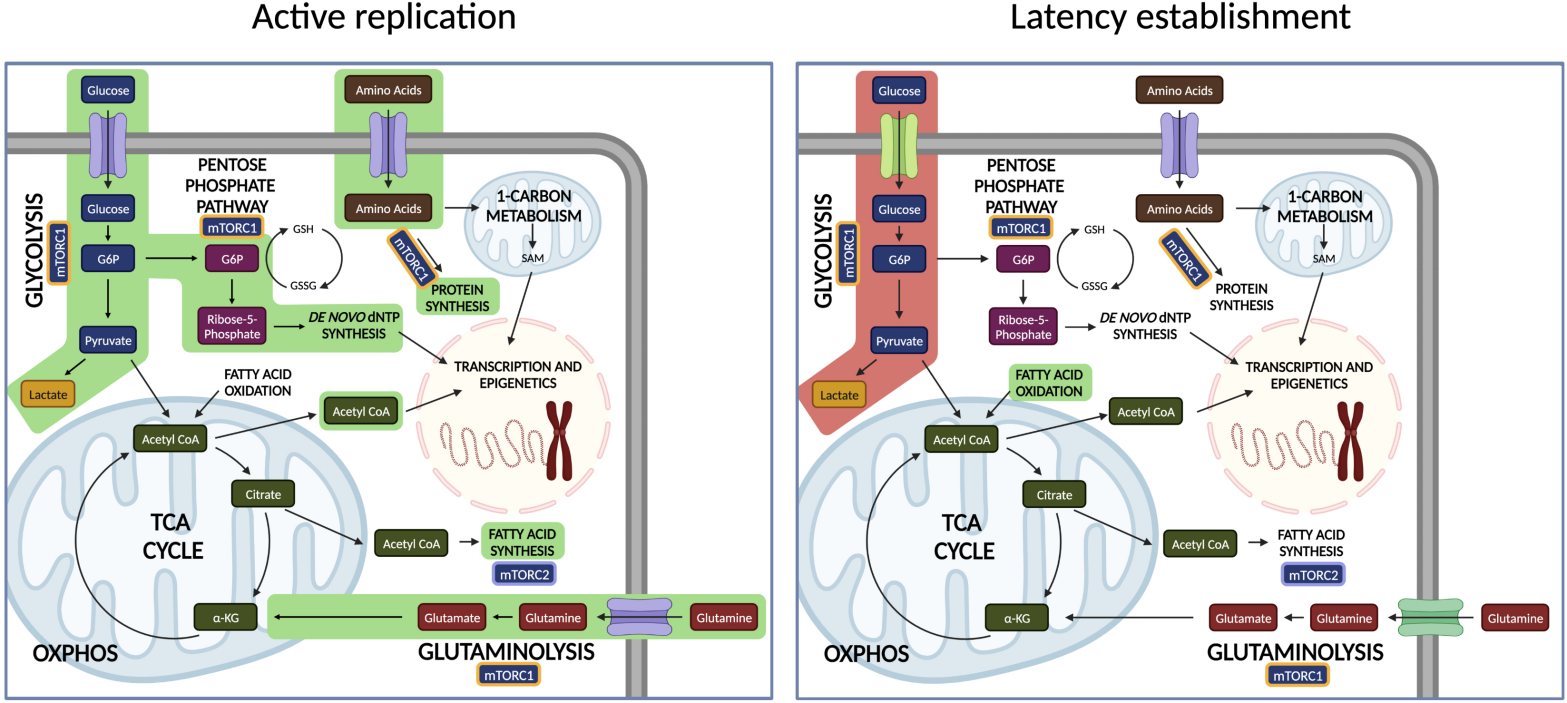
Metabolic pathways are differentially regulated in HIV-1 active replication and latent infection. During active replication, the infected cell must increase glucose and amino acid uptake to support normal anabolic functions in addition to virion production. During the establishment of latency, the cell progressively downregulates aerobic glycolysis. Decrease in mTORC1-dependent pathways reduces virions production. Pathways highlighted in green represent upregulation, while pathways highlighted in red represent downregulation.

The physiological metabolic shift that occurs following T cell activation and critical to productive HIV replication is partially driven by mTOR signaling. However, this shift is enhanced in HIV-1 infected cells compared to uninfected cells following activation (Hegedus et al., 2014), suggesting that glycolysis, glutamine utilization, TCA cycle/OXPHOS, and the PPP are intricately related to productive HIV-1 replication. The involvement of these four major metabolic pathways in HIV-1 replication is reviewed below **(** **Figure 1** **)**.

### Glycolysis and HIV-1 replication

Early reported correlations between HIV-1 replication and T cell activation led many investigators to investigate glycolytic activity and glucose uptake within HIV-1 infected cells. GLUT1 abundance on the cell surface is also an indirect measurement of glucose uptake and metabolic activity. Akt, downstream of both mTORC2 and PI3K, regulates GLUT1 surface abundance (Loisel-Meyer et al., 2012). Primary CD4^+^ T cells with increased GLUT1 surface levels are more susceptible to productive HIV-1 infection and replication (Valle-Casuso et al., 2019). This relates specifically to the activation state of the cell, as cells with a more active metabolic profile, such as effector memory cells, are more permissive to HIV-1 infection when compared to naïve or central memory T cells (Valle-Casuso et al., 2019) again showing the importance of glucose abundance and cellular glucose uptake in HIV-1 infectivity.

Aerobic glycolysis, or the Warburg effect, is used by highly proliferative cells like cancer cells or activated lymphocytes and can be measured by lactate abundance, LDH (lactate dehydrogenase) activity, or extracellular acidification of the culture medium. Although cells can completely oxidize glucose to carbon dioxide in the presence of oxygen, highly proliferative normoxic cells convert pyruvate into lactate in order to quickly replenish NAD^+^. HIV-1 further upregulates the already high glycolytic activity seen in activated T cells. The extracellular acidification rate (ECAR), a measure of glycolytic activity, of uninfected primary CD4^+^ T cells was increased 4-fold following addition of glucose compared to a 6-fold increase in HIV-1 infected cells (Hegedus et al., 2014).

Furthermore, inhibiting glycolysis with the competitive glucose-6-phosphate isomerase inhibitor, 2-DG, also reduces HIV-1 virion production (Hegedus et al., 2014; Valle-Casuso et al., 2019). To further support the link between glucose-dependent glycolytic steps and virion production, HIV-1 infected primary CD4^+^ T cells were cultured in either glucose or galactose containing media. Culturing cells with galactose alone, which is an inefficient substrate for glycolysis, caused p24 levels to drop 20% to 60% compared to glucose-containing media after 24 hours (Hegedus et al., 2014).

### Glutamine utilization and HIV-1 replication

In addition to upregulated glycolysis, HIV-1 replication requires cells to increase their uptake and utilization of glutamine and other amino acids (Valle-Casuso et al., 2019). This elevated glutamine utilization typically occurs following T cell activation, with an increase in the cell surface levels of the glutamine transporter ASCT2 (Clerc et al., 2019) and enzymes that metabolize glutamine. mTORC1 signaling is responsible for ASCT2 upregulation, and mTOR inhibition prevents ASCT upregulation upon CD3/CD28 stimulation of T cells (Taylor et al., 2020). Additionally, T cell activation increases the levels of GLS, the enzyme that converts glutamine to glutamate (Taylor et al., 2020). Increased glutamate production from glutamine can then be converted to α-KG and can be broken down further in the TCA cycle or used as a building block in multiple anabolic pathways.

Primary CD4^+^ T cell susceptibility to productive HIV-1 replication is correlated with glutamine availability (Clerc et al., 2019). Upon infection, intracellular glutamine levels increase, and virion production is correlated with increased glutamine availability (Hegedus et al., 2017). In HIV-1-infected primary CD4^+^ T cells, the majority of citrate comes from glutaminolysis as a source, rather than from glucose-derived pyruvate produced through glycolysis (Hegedus et al., 2017). Citrate can then be used to fuel the TCA cycle or can be broken down into acetyl-CoA for fatty acid synthesis or protein acetylation. Blocking glutamine utilization and glycolysis decreases the levels of productive HIV-1 infection and virion production.

### The tricarboxylic acid cycle, oxidative phosphorylation, and HIV-1 replication

While some reports have indicated that OXPHOS does not contribute significantly to virion production, other reports suggest OXPHOS may be more critical at different timepoints during viral replication. After HIV-1 infection of primary CD4^+^ T cells, the oxygen consumption rate (OCR), a measure of OXPHOS, was found to be decreased showing the reliance on glycolysis rather than OXPHOS (Hegedus et al., 2014). The addition of oligomycin, an ATP synthase inhibitor, to infected cells only minimally increased the ECAR, indicating that the cells were at their glycolytic capacity and therefore relying almost entirely on glycolysis during productive infection (Hegedus et al., 2014).

Although many studies highlight the importance of glycolysis for HIV production, OXPHOS may simply be more critical at different timepoints during the viral replication cycle and further analysis is warranted. The increase in mitochondrial and TCA enzymes during HIV replication does suggest that TCA/OXPHOS may still be important in HIV replication. After 24 hours post-infection, primary CD4^+^ T cells show increased amounts of TCA metabolites aconitate and isocitrate (Hollenbaugh et al., 2011). mTOR fuels the TCA cycle in two ways: through acetyl-CoA produced from increased glycolysis and through α-KG from glutaminolysis (Taylor et al., 2020). Mitochondrial function, necessary for both the TCA cycle and OXPHOS, is also supported by mTORC1 signaling (de la Cruz López et al., 2019).

Although glycolytic upregulation can be used as an indicator of permissivity to infection, Guo et al. has showed that OXPHOS is positively correlated during higher viral loads among PLWH without ARVs, an indicator of disease progression (Guo et al., 2021). OXPHOS upregulation was found in part to be due to the NLRX1 protein, which also positively correlated with HIV-1 viremia (Guo et al., 2021). In primary CD4^+^ T cells treated with metformin, an inhibitor of mitochondrial complex I, HIV-1 replication was suppressed, but not to the extent that 2-DG suppressed replication (Guo et al., 2021). Thus, while not the most critical factor in HIV virion production, the role of OXPHOS should not be overlooked.

### The pentose phosphate pathway and HIV-1 replication

The pentose phosphate pathway (PPP) plays a crucial role in maintaining cell physiology and homeostasis. In addition to creating 5-carbon sugars used in nucleotide synthesis, the PPP generates NADPH, which is required to maintain homeostatic levels of the antioxidant glutathione (GSH).

HIV-1 exploits the PPP to create a dNTP pool, as shown through increases of PPP intermediates in HIV-1 infected CD4^+^ T cells. Ribose 5-P and S7P levels increase in HIV-1 infected CD4^+^ T cells 24 hours post-infection (Hollenbaugh et al., 2011). In a separate study, HIV-1 infected CD4^+^ T cells were found to have decreased levels of glycolytic enzymes PGK and PGI three days post-infection (Geiss et al., 2000), indicating shunting of glucose through the PPP rather than glycolysis. Inhibiting mTOR in HIV-1 infected primary CD4^+^ T cells causes a decrease of PPP metabolites S7P, PRPP, and 6PG *via* SREBP signaling (Ben-Sahra and Manning, 2017; Taylor et al., 2020). This further shows the importance of mTOR activation in HIV-1 infection, as PPP activity is required for nucleotide synthesis and generation of lipids required for virion production (Lahouassa et al., 2012).

The pentose phosphate pathway also helps to maintain intracellular antioxidant concentrations and is important in reducing oxidative stress, which increases following HIV-1 infection (Sönnerborg et al., 1988). Oxidative stress refers to a state when reactive oxygen species (ROS), such as hydroxyl radicals or superoxide anions, overwhelm antioxidants. The production of ROS occurs naturally in the mitochondria from the electron transport chain during OXPHOS following T cell activation. However, ROS are highly reactive and can remove electrons from nucleic acids, proteins, and lipids. This requires cells to produce antioxidants that can homeostatically maintain oxidation within the cell. Through SREBP signaling, mTORC1 stimulates the oxidative branch of the PPP, in which NADPH is created, and increases the abundance of pentose phosphate metabolites (Duvel et al., 2010).

## Metabolic pathways affect the epigenetic control of HIV-1 latency and reactivation

Metabolic pathways regulated by mTOR influence chromatin states and HIV-1 transcription. Histone and DNA modifications which influence HIV-1 transcription and latency (Mbonye and Karn, 2014) are highly reliant on cellular metabolites (Su et al., 2016). Various HIV-1 cure strategies rely heavily on the epigenetics of the HIV-1 provirus, including the “kick and kill” and “block and lock” strategies (Ta et al., 2022). “Kick and kill” strategies attempt to reactivate latent provirus to make infected cells visible to the immune system. “Block and lock” strategies attempt to permanently silence the provirus epigenetically. Both cure strategies require a deep understanding of the epigenetic control of HIV-1 latency and reactivation. The variable epigenetic landscape of latently and productively infected cells is reliant on the cellular metabolic state (Shytaj et al., 2021).

mTOR and its downstream metabolic pathways regulate HIV-1 latency and reactivation (Besnard et al., 2016). During inititation of HIV-1 latency, glycolytic and PPP enzymes are progressively downregulated in primary CD4^+^ T cells (Shytaj et al., 2021). Enzymes downregulated included HK2, GPI, PFKL, ALDOC, TPI, and ENO2, the most extreme of which being GPI, which converts G6P to F6P (Shytaj et al., 2021). GPI downregulation commits glycolytic intermediates to the PPP rather than glycolysis. Upon reactivation of HIV-1, GPI levels increase, while cells less susceptible to reactivation have continued low levels of GPI expression (Shytaj et al., 2021).

Along with glucose, other metabolites affect viral latency and transcription. Amino acid uptake, fatty acid oxidation, and the TCA cycle all contribute to the intracellular acetyl-CoA pools and one-carbon metabolism. These are essential for histone acetylation, protein and DNA methylation and subsequent proviral expression. The epigenetic modifications of this section will primarily focus on acetylation and methylation, both of which are under the control of mTOR, as will be discussed next.

### Acetyl-CoA levels and HIV-1 replication

HIV-1 infection increases acetyl-CoA abundance through both increased glucose and glutamine transport mediated *via* Akt and mTOR signaling (Hegedus et al., 2014; Clerc et al., 2019; Taylor et al., 2020). The acetylation of histones is dependent on the availability of acetyl-CoA (Sivanand et al., 2018), a central metabolite in multiple metabolic pathways (Sivanand et al., 2018). An abundance of acetyl-CoA can lead to broad histone acetylation of lysine residues by histone acetyltransferases (HATs), generally promoting gene transcription (Su et al., 2016). HAT expression has also been found to be increased four-fold in whole blood RNA from untreated PLWH compared to seronegative individuals (Marimani et al., 2020). In contrast, histone deacetylase (HDAC) mRNA was only found to be increased two-fold (Marimani et al., 2020).

While the activity of all HATs is dependent on acetyl-CoA availability, class III histone deacetylases (HDACs), known as sirtuins, additionally require NAD^+^ as a cofactor. As sirtuins are NAD^+^ dependent, they are under metabolic control *via* NAD^+^ availability, providing yet another way that host cell metabolism affects HIV-1 transcription. NAD^+^ is consumed during glycolysis and the TCA cycle and is created through various metabolic sources, including OXPHOS and the production of lactate *via* LDHA in aerobic glycolysis, which is upregulated by mTOR (Taylor et al., 2020).

The role of sirtuins linking metabolism and HIV transcription needs further investigation. Sirtuin 1 has been described to increase HIV 1 transcription in cell lines (Pagans et al., 2005) but conflicting results have been reported about SIRT1 (Sirtuin 1) mRNA in the PBMC of PLWH. SIRT1 mRNA has been reported to be decreased by Gruevska et al. (Gruevska et al., 2022) but reported to be increased by Bogoi et al. (Bogoi et al., 2018), indicating that further investigation is needed.

### One-carbon metabolite levels and HIV-1 replication

Methylation can occur on both histones and DNA using one-carbon metabolites. Histone methylation can either promote or silence gene expression depending on the residue while DNA methylation at CpG sites are typically inhibitory. The methyl donor for histone and DNA methylation, S-Adenosylmethionine (SAM), is created through one-carbon metabolism, which comprises the folate and methionine cycles (Chisolm and Weinmann, 2018).

Metabolism and epigenetics are interdependent. Amino acids such as serine, glycine and threonine are substrates for one-carbon metabolism (Locasale, 2013). mTORC1 regulates SAM abundance through Myc signaling (Villa et al., 2021). Both DNA and histone demethylases require α-KG as a cofactor to perform their catalytic functions (Chisolm and Weinmann, 2018), thus relying on the TCA cycle or glutaminolysis. Demethylase complexes are inhibited by succinate, fumarate, and 2-HG (Chisolm and Weinmann, 2018).

HIV-1 infection results in changes in the levels of enzymes involved in one-carbon metabolism and downstream methylation and demethylation reactions. HIV-1 infection and mTOR activation increase levels of the one-carbon metabolism substrate, serine, through upregulating the amino acid transporter ASCT2 (Taylor et al., 2020). When comparing the CD4^+^ T cells from PLWH to those from uninfected individuals, there was shown to be a 4-fold increase in the histone methyltransferase SETDB1 in cells from PLWH (Bogoi et al., 2018). SETDB1 has an inhibitory role in gene expression through its interactions with DNMT3A and HDAC1, which were also seen to be upregulated (Bogoi et al., 2018). This may be involved in the establishment of latency. Another histone methyltransferase, PRMT6, was found to be decreased 3-fold in HIV-1 infected samples. PRM6 methylates the HIV-1 protein Tat in an inhibitory manner (Boulanger et al., 2005). These histone methyltransferase enzymes are all, of course, dependent on the availability of SAM as produced through one-carbon metabolism and amino acid uptake regulated by mTOR.

DNA methylation has also been implicated in the silencing of the HIV-1 provirus and the establishment of latency. The methyl-CpG binding protein MBD2 was upregulated 2-fold in the CD4^+^ T cells from PLWH. This was found in addition to higher levels of genome methylation in HIV-1 infected CD4^+^ T cells when compared to negative controls (Bogoi et al., 2018). Quantifying the methylation of CpG sites in the HIV-1 5’-LTR of latently infected CD4^+^ T cells, Blazkova et al. determined that aviremic elite controllers had between 20-100% methylation of CpG sites while viremic individuals showed less than 0.1% CpG methylation, exhibiting the strength of CpG methylation in proviral suppression (Blazkova et al., 2009). Blazkova et al. then attempted to reactivate the virus from the CD4^+^ T cells from PLWH with TNF-α + PMA or TSA + PMA in addition to the presence of IL-2 for three days, and it was subsequently found that there was a negative correlation between CpG methylation and the ability to reactivate the provirus (Blazkova et al., 2009). It was noted that two of the CD4^+^ T cell cultures from HIV-1-infected aviremic individuals were reactivated from latency, but they were found to contain fewer methylated promotors than cell cultures that retained latency from aviremic infected individuals (Blazkova et al., 2009). In contrast, all CD4^+^ T cell cultures from viremic individuals underwent latency reversal after stimulation (Blazkova et al., 2009). Corroborating the importance of DNA methylation in HIV-1 latency, Yang et al. found that knocking out methionine adenosyltransferase 2A (MAT2A) in the J-Lat 9.2 cell line led to HIV-1 latency reversal (Yang et al., 2021). MAT2A creates SAM (S-Adenosylmethionine), the methyl donor in histone and DNA methylation, indicating the importance of SAM for maintenance of latency. MAT2A can be activated by Myc signaling, downstream of mTORC1 (Villa et al., 2021). Yang et al. further investigated the HIV-1 LTR in the MAT2A knockout and showed decreased CpG island methylation by approximately 50% as well as decreased methylation of histones around the HIV-1 LTR, such as H3K4me3, H3K9me3, and H3K27me3 (Yang et al., 2021). To validate their findings, Yang et al. used primary CD4^+^ T cells treated with SAM to increase methyl availability and silencing of the HIV-1 genome. After treatment with two latency reversing agents, PMA and ionomycin, latency persisted, indicating the strength of methylation in maintaining latency (Yang et al., 2021). These findings show the role of one-carbon metabolism and methyl group availability in the epigenetic control of HIV-1.

Although epigenetics is increasingly studied in the HIV field, this avenue of investigation is still young. There are many unanswered questions regarding epigenetic controls of HIV transcription and latency; however, to answer some of these questions, it will be important to take into account host cell metabolism, which is highly dependent on the regulatory properties of mTOR.

## HIV-1 proteins utilize host cell metabolism to fuel the viral replication cycle

### Metabolism and HIV-1 entry

Showing that T cell activation is closely tied to HIV infectivity and virion production, Valle-Casuso et al. expanded on studies reminiscent of research in the 1980s which focused on T cell activation and HIV-1 (Valle-Casuso et al., 2019). However, Valle-Casuso’s work greatly extended beyond the previous principles from decades before, expanding to how HIV-1 infection changes cell metabolism within different activation phenotypes. In 2019, Valle-Casuso et al. reported that metabolic profiles of CD4^+^ T cells are closely related to susceptibility of cells to HIV-1 entry and replication. Naïve CD4^+^ T cells, the most metabolically quiescent CD4^+^ T cell subtype, were found to be relatively resistant to HIV infection. Transitional and central memory T cells were slightly susceptible to HIV-1 infection, and effector memory CD4^+^ T cells, the most metabolically active T cell subset, showed the highest levels of HIV-1 infection (Valle-Casuso et al., 2019). To test the effect of activation on HIV-1 infectability between subsets, cells were activated and challenged with NL4.3ΔenvGFP to ensure single-round infection. Activation increased the susceptibility to infection in all T cell subtypes, but the increase in infection was most pronounced in T effector memory cells, while only a minor difference was detected in naive T cells. Infectivity was also blocked by inhibiting glycolysis with 2-DG (Valle-Casuso et al., 2019). The most dramatic decreases in virus infectivity were seen in effector memory T cells, the most metabolically active CD4+ T cell subset (Valle-Casuso et al., 2019). While higher metabolic rates strongly correlate with infectability, this shows that all T cells memory subset can be infected by a single-round HIV 1 virus *in vitro*.

### Metabolism and HIV-1 reverse transcription

A crucial determinant of reverse transcription of HIV-1 is the availability of dNTPs. The retroviral restriction factor, SAMHD1, prevents reverse transcription affects viral infectivity in monocyte derived macrophages and CD4^+^ T cells by reducing the intracellular pool of dNTPs (Lahouassa et al., 2012; Baldauf et al., 2012). The creation of new dNTPs is dependent on PRPP, a product of the pentose phosphate pathway. Inhibition of mTOR in primary CD4+ T cells decreases PPP metabolites S7P, 6PG, and PRPP (Taylor et al., 2020).

### Metabolism and proviral transcription

HIV-1 Tat plays a major role in transactivating proviral transcription at the LTR. The main function of Tat is supporting the elongation of proviral transcription (Rice, 2017), but Tat has also been found to increase reactive oxygen species. Primary CD4^+^ T cells infected with HIV-1 show markers of oxidative stress, such as a decreased GSH/GSSG ratio (Shytaj et al., 2020) and this elevated ROS may enhance LTR activation (Schreck et al., 1991). However, in primary CD4^+^ T cells, HIV-1 Tat was also found to be necessary in the upregulation of G6PDH expression, the first enzyme in the PPP (Shytaj et al., 2021), which is important in increasing cellular antioxidants. Thus, Tat may influence both the increase of ROS linked to HIV-1 LTR transcription as well as the induction of pathways that remove ROS as an infected cell progresses to latency.

### Metabolism and HIV-1 assembly and budding

The reliance of HIV-1 assembly and budding on host-cell metabolism is largely related to the viral mRNA and protein synthesis along with the availability of lipids such as cholesterol.

After proviral transcription, translation occurs in the cytoplasm, after which viral assembly can occur. The newly transcribed Gag polyprotein, which will later be cleaved into the matrix, capsid, nucleocapsid, and p6 proteins, travels with HIV-1 RNA to the cell membrane (Freed, 2015). To begin the viral assembly process, Gag binds to a cholesterol-enriched lipid raft on the cell membrane (Ono et al., 2007). Lipid rafts are areas on the cell membrane that are enriched in sphingolipids and cholesterol and are used as a site for vesicle budding or where transmembrane proteins may be located (Simons and Sampaio, 2011). The availability of lipids to be used in lipid rafts is therefore important in both normal cellular function and the HIV-1 life cycle. The depletion of intracellular cholesterol decreases the amount of Gag able to bind to the plasma membrane (Ono et al., 2007). The binding of Gag to the membrane also causes lipid raft components to further aggregate (Hogue et al., 2011).

In addition to the transcriptional and translational enhancing activities of mTOR, the mTORC1 complex enhances lipid synthesis through SREBP signaling (Ben-Sahra and Manning, 2017). This signaling is increased in productively infected CD4^+^ T cells (Taylor et al., 2020). Primary CD4^+^ T cells rely on mTOR signaling during activation to increase fatty acid uptake (Angela et al., 2016). S6 phosphorylation, an indicator of mTOR activity, has been found to positively correlate with fatty acid uptake in primary CD4^+^ T cells (Angela et al., 2016). Furthermore, mTOR inhibition with rapamycin decreased PPARγ mRNA and SREBP1 expression, both of which are important for fatty acid uptake (Angela et al., 2016). Inhibiting PPARγ also prevented robust CD4^+^ T cell activation (Angela et al., 2016) which is necessary for HIV-1 infection (Valle-Casuso et al., 2019).

## Conclusion

Ongoing HIV-1 research focused on host cell metabolism has extended in many different directions, including cure strategies that involve mTOR signaling. Multiple pre-clinical studies have been done using rapamycin or rapalogs in mice and nonhuman primates (NHP). In humanized mice, ATP-competitive mTOR kinase inhibitors were used to show a protective effect of mTOR inhibition on HIV infection (Heredia et al., 2015). This is likely partially due to the inhibitor-induced decrease of CCR5 expression on target cells. In NHP studies, rapamycin was shown to reduce proliferation of memory CD4^+^ T cell subsets. Although clonal proliferation of provirus in this latent reservoir subset should be reduced, the authors were unable to measure any decrease in the latent SIV reservoir following rapamycin treatment compared to controls (Varco-Merth et al., 2022). Additionally, studies evaluating the efficacy of Akt/mTOR activation as a possible latency reversing agent (LRA) have had little *in vivo* success (Gramatica et al., 2021).

Pharmacological derivatives of rapamycin, or rapalogs, that inhibit mTOR complexes include tacrolimus, sirolimus, and everolimus have been used in multiple clinical studies involving PLWH (Henrich et al., 2021; Diaz et al., 2021). Although cell-associated HIV-1 DNA and RNA levels did not change in the full cohort, participants who maintained increased levels of everolimus in the first two months also had decreased RNA levels for at least the first six month following removal of the drug (Henrich et al., 2021). Drug-drug interactions between antiretrovirals and mTOR inhibitors have to be closely monitored to prevent toxicity and adverse events (Diaz et al., 2021). Other studies have investigated indirect mTOR inhibitors such as metformin to show that there were no changes in the HIV transcriptionally-competent reservoirs found in peripheral blood CD4^+^ T cells. However, in this study, metformin was shown to reduce mTOR activation and HIV transcription in colon memory CD4^+^ T cells in a subset of ART-treated PLWH (Planas et al., 2017; Planas et al., 2021). In future clinical studies, it will be imperative to take both age and sex into consideration since both inflammation and metabolism are highly regulated by these two factors.

**Figure 2:**
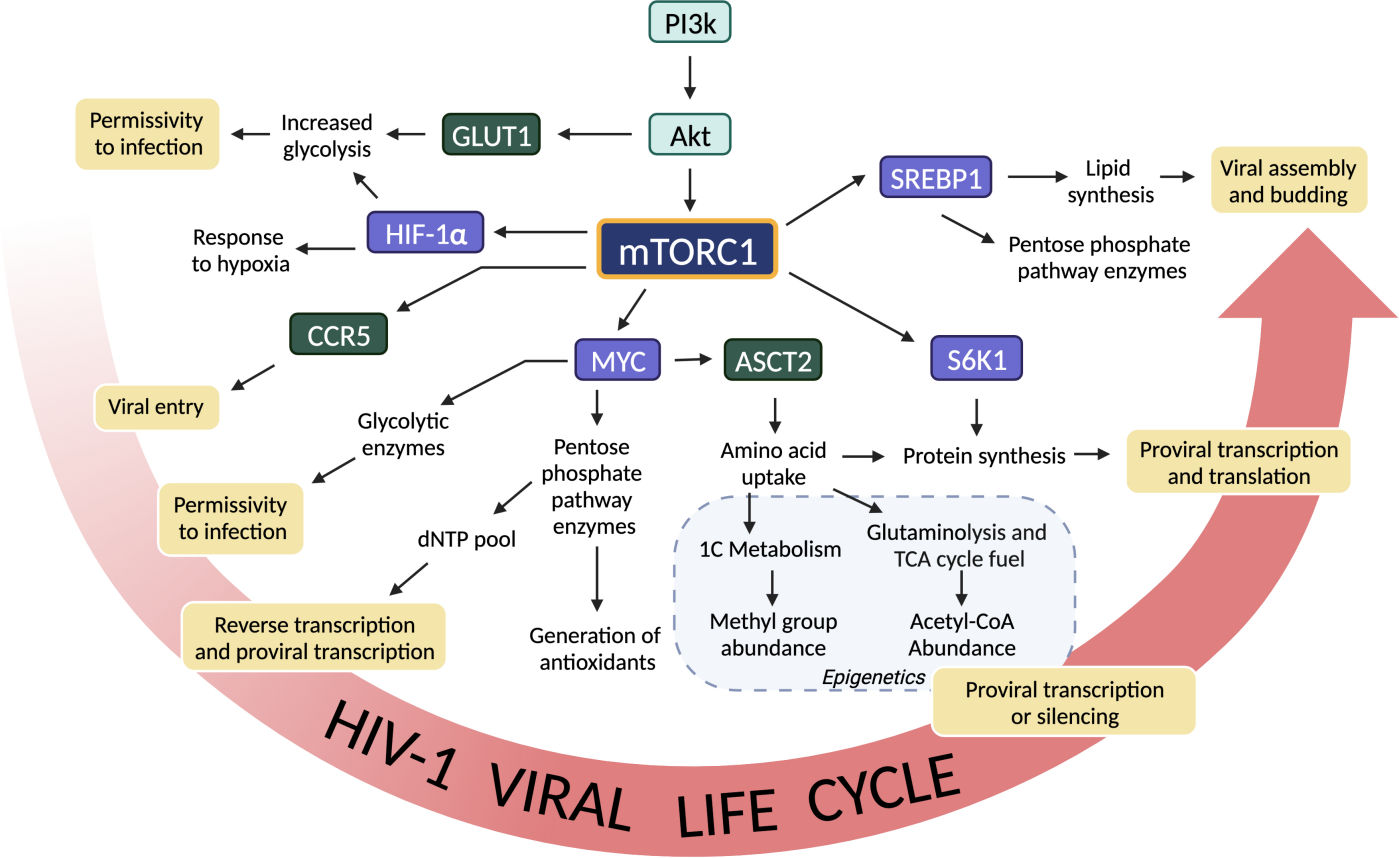
mTORC1 is involved of each step of the HIV-1 viral life cycle. Factors downstream of mTORC1 lead to increased permissivity to infection, reverse transcription, proviral transcription, epigenetic control of the provirus, protein translation, virion assembly, and budding. mTORC1 affects these stages of the viral life cycle by regulating signaling and metabolic pathways. Kinases upstream of mTORC1 are shown in light blue, surface proteins are shown in green, and transcription factors are shown in purple.

Each stage of the HIV replication cycle is dependent on mTOR-driven metabolic pathways (**Figure 2**). Although the importance of cellular metabolism in regulating HIV-1 replication and latency has increased in recent years, this insight is merely another way to describe something that has been known for decades. HIV-1 replication is dependent on the activation state of the host CD4^+^ T cell. This “activation state” is driven by changes in cellular metabolism and will be difficult to manipulate specifically in target cells (infected CD4^+^ T cells) without simultaneously altering critical metabolic pathways in bystander and effector CD8^+^ T cells. Although rapalogs and other mTOR inhibitors have not shown efficacy in reducing the viral reservoir *in vivo* to date, the importance of cell metabolism in HIV replication and latency should not be overlooked when developing new HIV-1 cure strategies. Cellular metabolism is a critical component of understanding both physiological CD4^+^ T cell function and HIV-1 replication.

## Author contributions

JC, DN, and RFO wrote and edited the manuscript. All authors contributed to the article and approved the submitted version.

## Funding

This publication resulted in part from research supported by National Institute of Allergy and Infectious Diseases (NIAID) award UM1 AI126617, co-funded by National Institute on Drug Abuse (NIDA), National Institute of Mental Health (NIMH), and National Institute of Neurological Disorders and Stroke (NINDS), by award UM1AI164565 REACH, co-funded by NIMH, NIDA, NINDS, NIDDK, and NHLBI, and award R21AI154956 (NIAID).

## Conflict of interest

The authors declare that the research was conducted in the absence of any commercial or financial relationships that could be construed as a potential conflict of interest.

## Publisher’s note

All claims expressed in this article are solely those of the authors and do not necessarily represent those of their affiliated organizations, or those of the publisher, the editors and the reviewers. Any product that may be evaluated in this article, or claim that may be made by its manufacturer, is not guaranteed or endorsed by the publisher.
